# Mechanically Interlocked Molecular Rotors on Pb(100)

**DOI:** 10.1021/acs.nanolett.4c05409

**Published:** 2025-01-13

**Authors:** Chao Li, Yan Lu, Ruoning Li, Li Wang, Alexander Weismann, Richard Berndt

**Affiliations:** † Institut für Experimentelle und Angewandte Physik, Christian-Albrechts-Universität, 24098 Kiel, Germany; ‡ Department of Physics, Nanchang University, Nanchang 330031, People’s Republic of China; ¶ CAS Key Laboratory of Molecular Nanostructure and Nanotechnology, CAS Research/Education Center for Excellence in Molecular Sciences, Beijing National Laboratory for Molecular Sciences (BNLMS), Institute of Chemistry, Chinese Academy of Sciences, Beijing 100190, People’s Republic of China

**Keywords:** Molecular Rotors, Molecular Manipulation, Scanning Tunneling Microscopy, Molecular Interactions at Surface, Phthalocyanines

## Abstract

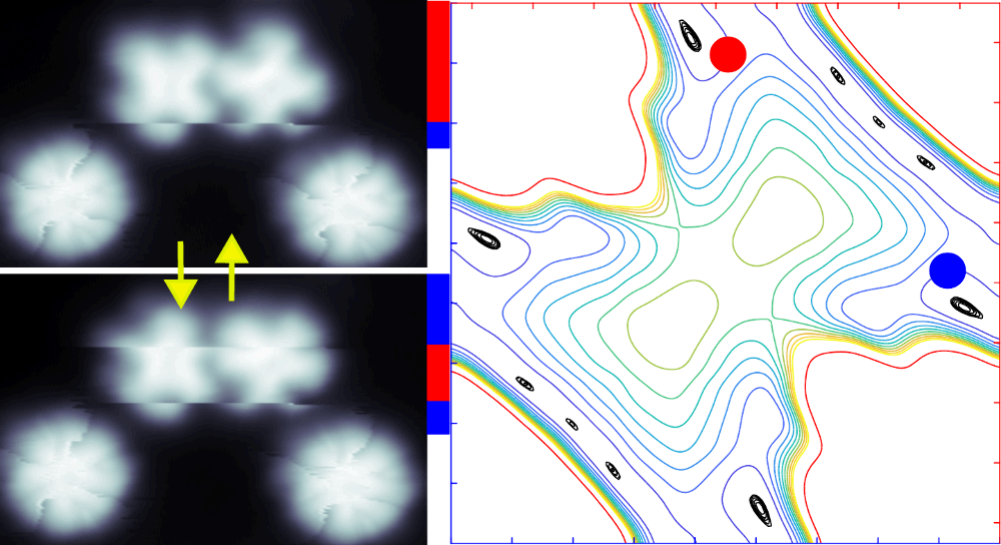

The mechanical coupling between molecules represents a promising route for the development of molecular machines. Constructing molecular gears requires easily rotatable and mutually interlocked pinions. Using scanning tunneling microscopy (STM), it is demonstrated that aluminum phthalocyanine (AlPc) molecules on Pb(100) exhibit these properties. Unlike other phthalocyanines on this substrate, isolated AlPc molecules fluctuate between two azimuthal orientations. Density functional theory (DFT) calculations confirm two stable orientations of single molecules and indicate a relatively low rotation barrier. In STM-constructed dimers and trimers, fluctuations diminish, and various molecular orientations are stabilized. Induced collective rotation of all molecules in the trimers is observed, demonstrating their mechanical interlocking. Potential functions describing angle and distance dependencies of intermolecular and molecule–substrate interactions are derived from DFT calculations of dimers; 52 experimentally determined trimer geometries are reproduced using these potentials. This intuitive approach may prove to be useful in modeling larger structures beyond the scope of quantum mechanical descriptions.

The development of artificial machines that control mechanical motion at the molecular level has the potential to impact a range of fields.^1,2^ Recently, research has focused on the use of scanning tunneling microscopy (STM) to study simple molecular machines, particularly molecular rotors, mounted on surfaces.^3−25^ While the mechanical interlocking of molecules plays an important role in transferring motion between different parts of a machine,^26^ there has been little imaging and operating of mechanical gear mechanisms using scanning tunneling microscopy.^17,18,20^ The quantum mechanical modeling of interactions in such machines is impeded by the dimensions of the relevant structures. However, single molecules acting as gears have been studied with density functional theory.^27^ Molecular dynamics calculations of arrays have also been reported and used to derive an interaction potential.^28^

In this study, we present scanning tunneling microscopy (STM) data on the interactions between AlPc molecules in artificial arrays on Pb(100). Our observations indicate that isolated AlPc molecules on this surface exhibit rotational motion when imaged by STM at 4.2 K. However, embedding these molecules into artificial dimers and trimers may serve to stabilize their orientations. Additionally, we have observed the transfer of rotational motion from one end of a trimer to the other. We combine experimental data from various dimer configurations with density functional theory (DFT) calculations to derive pair potentials for intermolecular interactions. These potentials are employed to analyze the molecular distances and azimuthal orientations within trimers. The resulting model geometries show agreement with many observed trimers, demonstrating that the relatively simple approach effectively captures key aspects of the data. While ab initio modeling of larger artificial arrays of molecules is currently beyond reach, the use of interaction potentials proves highly effective for the system studied here and may offer a practical and insightful framework for analyzing other molecular systems.

## Mobility of Isolated Molecules

**Figure 1:**
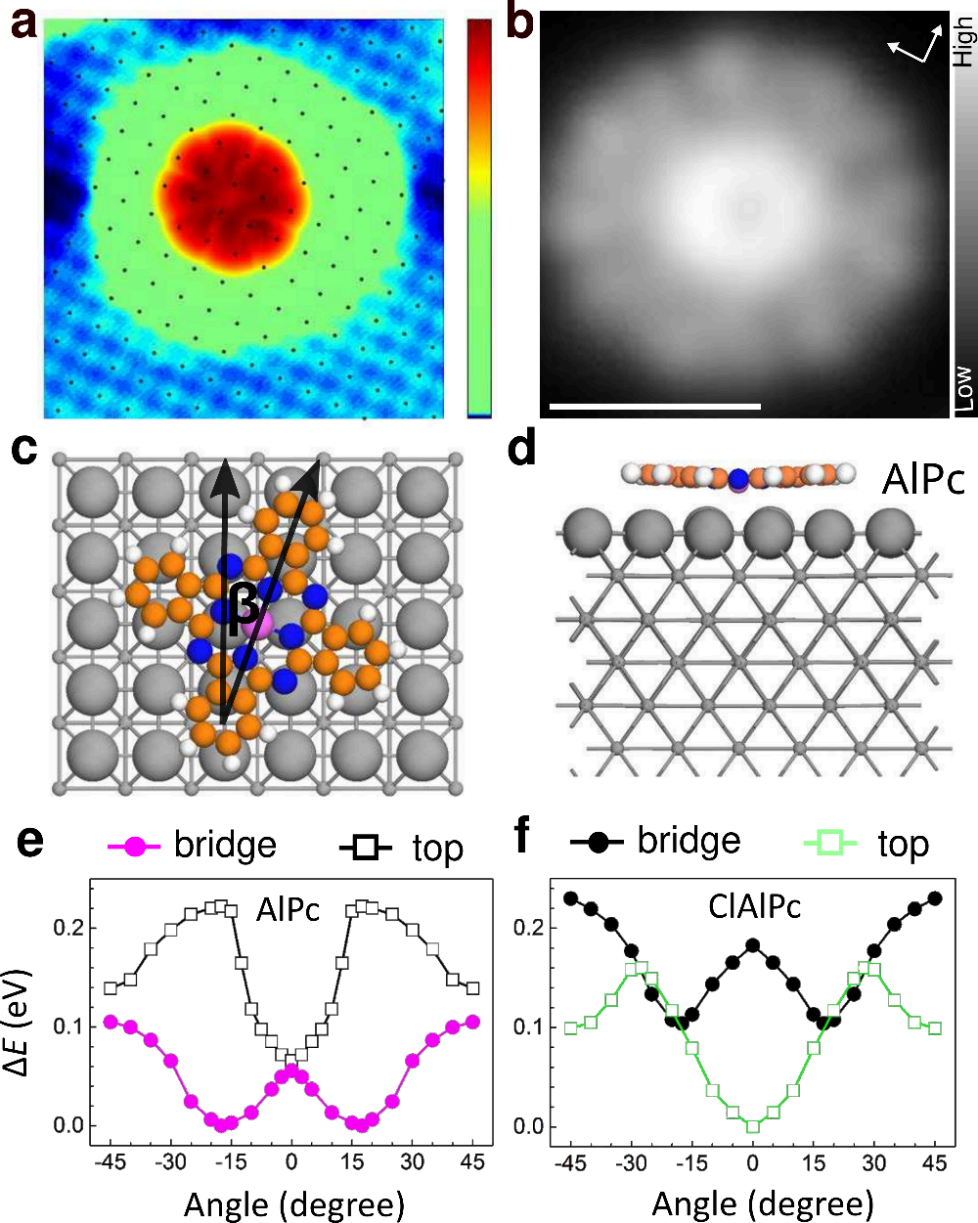
**Isolated AlPc molecule on Pb(100). a**, Atomically resolved STM image of an isolated AlPc molecule (red) on Pb(100). Black dots indicate substrate atoms (*V* = 10 mV, *I* = 1 nA, 4.5 × 4.5 nm^2^, height range 175 pm). The greenish halo around the molecule is due to the shape of the STM tip. **b**: Constant height image of an AlPc molecule recorded over a period of 35 min. The averaging of different orientations results in a pattern with 16 lobes (*V* = 300 mV). A white bar corresponds to 1 nm. The arrows indicate ⟨110⟩ directions of the substrate. **c, d** Top and side views of the geometry of AlPc on Pb(100) from DFT calculations. The gray, orange, blue, magenta, and white dots indicate Pb, C, N, Al, and H, respectively. **e, f**: Change of total energies vs molecular orientation for AlPc (**e**) and ClAlPc (**f**) on top (squares) and bridge (dots) sites. The lowest-energy configurations are used to define Δ*E* = 0.

AlPc molecules, which are unstable in the gas phase, have recently been prepared on Pb.^29^ In contrast to H_2_Pc and PbPc on the same surface^30,31^ AlPc is unstable in STM images, which leads to blurred image contrast (red area in Figure 1a) rather than four or eight well-defined lobes. Constant-height imaging over an extended time interval (35 min) resulted in Figure 1b showing a pattern of 16 blurred lobes. We attribute the image contrast to time averaging over fluctuations between two orientations of the molecule. A similar instability has previously been reported from measurements at room temperature, 80 K,^14,32^ and 7 K in the case of CuPc on Cu(111).^9,10^ It appears that the energy barrier for the molecular rotation of AlPc on Pb(100) is particularly low.

The image in Figure 1a also resolves the square substrate lattice (black dots) and shows that AlPc adsorbs at the bridge sites of the Pb surface. This is different from the top site adsorption of H_2_Pc and PbPc.^30,31^

Our DFT calculations of isolated AlPc on Pb(100) reveal the variation Δ*E* of the total energy with the angle β between the molecular isoindole lobes and a substrate ⟨110⟩ direction (Figure 1c). Δ*E* was also calculated for ClAlPc-up and PbPc-up (Figure 1f and Supporting Figure S1). These molecules adsorb at top sites without discernible fluctuations.^33^ Δ*E* differs greatly between top and bridge adsorption sites. For top adsorption, minima occur at β = 0° and 45° matching the adsorption geometries of H_2_Pc and PbPc. For bridge adsorption, i.e. AlPc, however, the potential curves are inverted, and minima are located at β ≈ ±18°. As will be shown below, these angles agree with the experimental data for AlPc.

The rotation barrier separating the minima of 56 meV for AlPc is lower compared to those of PbPc and ClAlPc, which may explain why AlPc fluctuates between two orientations in the STM. Unfortunately, the uncertainty of this value is significant. The calculated barrier height varied between 17 and 56 meV depending on the number of Pb layers and the van der Waals functional used. The calculations also show that isolated AlPc molecules, which have an unpaired electron in the gas phase, gain an electron on Pb(100) and become nonmagnetic.

It is worth noting that our preliminary calculations with a six-layer Pb slab indicated a preference for the top site adsorption of AlPc (Supporting Figure S1). For an 11-layer slab, the experimentally determined sites of AlPc, PbPc, and ClAlPc are reproduced (Supporting Figure S2). This sensitivity to the slab thickness is presumably attributable to quantum size effects, which are pronounced in Pb slabs.^34^ DFT further confirms that the energy barrier for shifting a molecule to neighboring bridge sites through intermediate hollow or top sites is higher than the rotation barrier (Supporting Figure S3).

### Interacting Molecules in Artificial Arrays

**Figure 2:**
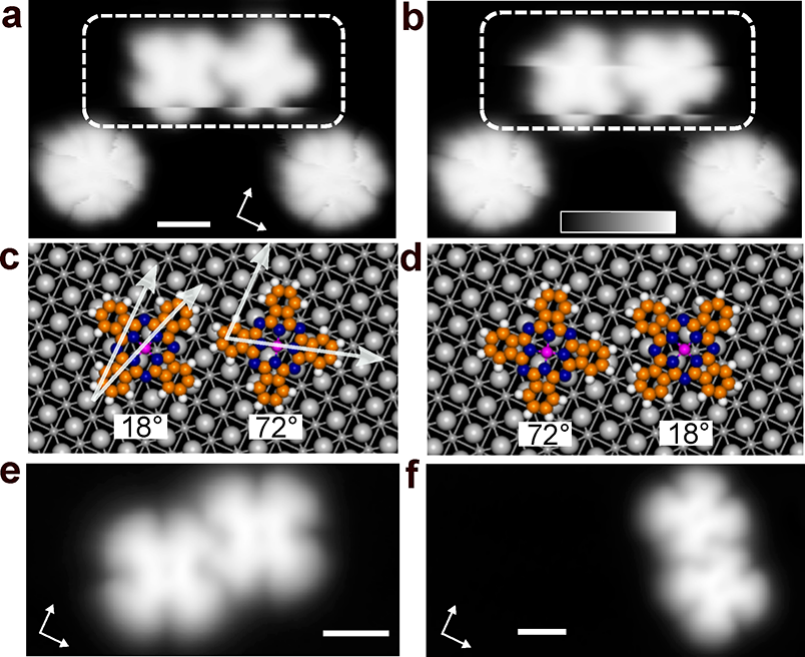
**AlPc dimers on Pb(100).** (**a**, **b**): Constant-current images of dimers with an intermolecular distance of 1.57 nm and isolated molecules. Acquisition time per image 4 s, time lapse between the images 1 min. While the isolated molecules appear almost circular owing to frequent changes of their orientations, the molecules of the dimer are more stable and undergo only few collective rotations, which lead to horizontal streaks in the images (*V* = 10 mV, *I* = 100 pA). (**c**, **d**): Models of the dimers in **a** and **b**. The angle β between the molecular isoindole lobes and a substrate ⟨110⟩ direction is indicated. (**e**, **f**): Dimers with 1.49 nm separation, imaged at the same conditions. The molecular orientations are stable with angles β = ±18°, where −18° is symmetry equivalent to 72°.

**Figure 3:**
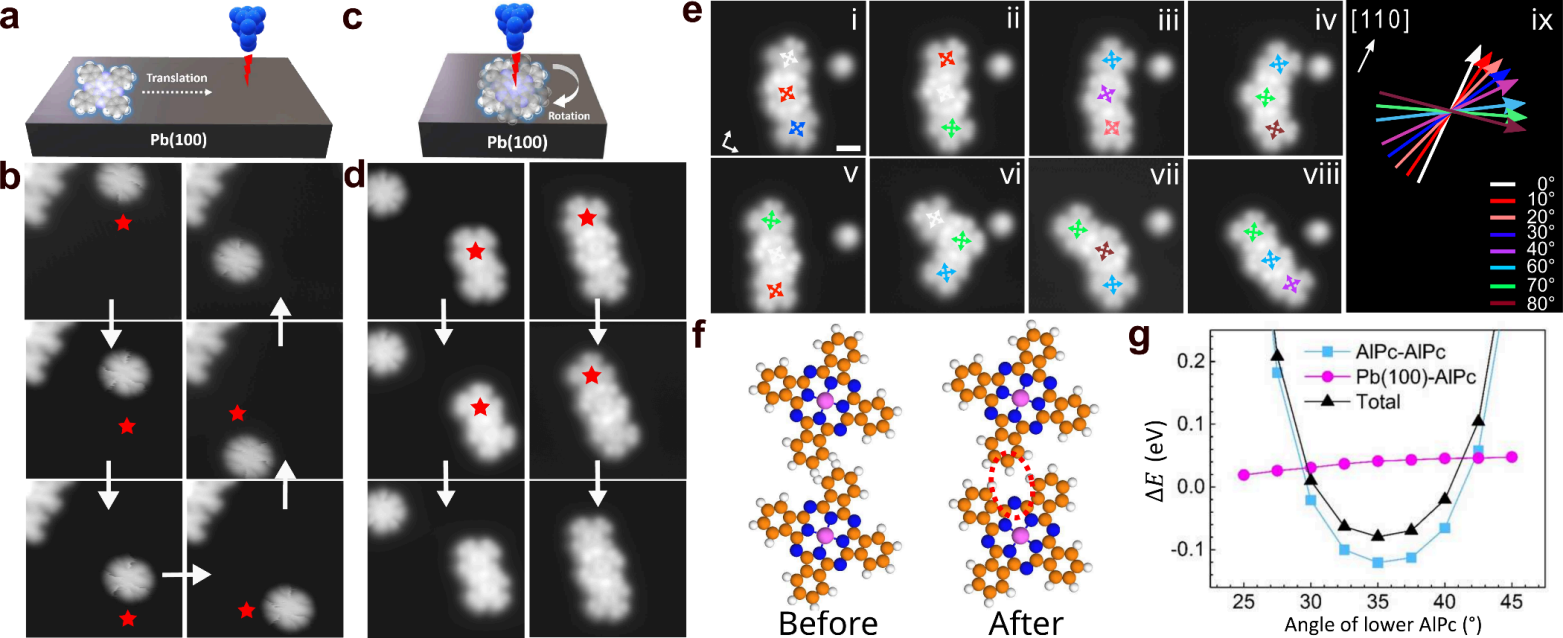
**Mechanical interlocking of molecules. a**: Lateral manipulation using voltage pulses. A 2 V-pulse applied to a location at a nm-distance from the molecule causes it to move below the STM tip. **b** Pulses applied at positions indicated by red stars induce lateral motion (imaging at *V* = 10 mV, *I* = 100 pA). **c**: A 2 V-pulse applied with the tip positioned above the center of the molecule induces molecular rotation. **d**: A pulse applied to the molecules indicated by the red star in mechanically interlocked dimers and trimers results in collective rotation. Another pulse can be employed to revert to the original orientations (*V* = 100 mV, *I* = 100 pA). **e**: In different trimers, additional distinct molecular orientations are observed. In addition to β = ±18°, the following values were obtained: 0, 10, 20, 30, 40, 60, 70, and 80° as indicated by colored crosses (*V* = 100 mV, *I* = 100 pA). **f**: Models of an adsorbed dimer with *v⃗* = [0, −4]. Left: Initial geometry with β = 18° for both molecules. Right: Relaxed structure obtained by DFT calculations. **g**: The change in total energy from DFT calculations (black triangles) is plotted against the angle β of the lower molecule. The upper molecule alters the optimal orientation of the lower molecule from β_2_ = 18 to 35°. The variation of the intermolecular interaction (cyan squares) is significantly greater than that of the molecule–substrate interaction (magenta circles).

STM-induced translation of molecules was possible by applying voltage pulses at a target position on the substrate near an isolated molecule (Figure 3a).^35−38^ Such a pulse reproducibly moved the molecule to the desired position, as shown in a sequence of induced translations in Figure 3b. This technique was used to construct dimers and trimers. Figure 2 shows an example. The dimer depicted in panels a and b (dashed rectangle) is comprised of molecules with a center-to-center distance *d* = 1.57 nm. It fluctuates less often than the isolated molecules in the same images, and its molecular orientations are resolved. The four-lobe patterns of the stabilized molecules are oriented at β = ±18°, in agreement with the calculated result. Whenever fluctuations occur (once in Figure 2a, twice in b), both molecules collectively rotate in opposite directions so that the relative tilt between both molecules is always ≈36°.

Below, the dimer geometry is described by the vector *v⃗* connecting the centers. Its components are given in units of the Pb nearest neighbor distance (*a* = 350 pm) along the [110] and [1̅10] directions. The dimer of Figure 2a has *v⃗* = [4, 2]. Figure 2e and f show dimers with a reduced distance *d* = 1.49 nm = *a*|*v⃗*| with *v⃗* = [3, ±3]. Here both molecules were stabilized to identical angles of β = ±18°; no tilt between the molecules was present, and fluctuations were absent over the duration of the measurement.

Taken together, the data indicate that isolated AlPc molecules adsorb with β = ±18°, but the energy barrier between these states is low enough to allow for rotation during scanning. The close proximity of a second molecule prevents the motion, while collective rotation remains possible at an intermediate distance.

A rotation can be induced in a controlled manner by applying a voltage pulse above the molecular center (Figure 3c). The image sequences in Figure 3d show experimental examples. Pulses were applied to the center of the molecules marked by stars. This led to rotation of all molecules in a dimer and a trimer (middle row), indicating that the molecules are mechanically interlocked. A second pulse above the topmost molecules (middle row, stars) turned all molecules to their initial orientations (bottom row). Attempts to extend the controlled collective rotation to linear tetramers failed.

A large variety of trimers were prepared. Examples are displayed in Figure 3e, panels i–viii, with colored crosses indicating molecular orientations. The corresponding displacement vectors *v⃗*_*ij*_ between molecules *i* and *j* and the molecular orientations are summarized in Table 1. The molecular orientations β in trimers are stable and often deviate from the 18° observed in dimers. Even values close to β = 45° have been observed where a maximum of the molecule–substrate potential is located. In total, eight orientations in steps of 10 ± 5° can be discerned as indicated schematically in panel ix. This remarkable variability of the orientation shows that the intermolecular interaction controls β, while the AlPc-substrate interaction appears to have little effect on the in-plane orientation of the molecules.

**Table 1:** Structural Parameters of Trimers: Experiment vs Model^a^

						Model											
	Experiment					State 0			State 1			State 2			State 3		
Trimer	*v⃗*_12_	*v⃗*_23_	β_1_	β_2_	β_3_	β_1_	β_2_	β_3_	β_1_	β_2_	β_3_	β_1_	β_2_	β_3_	β_1_	β_2_	β_3_
i	[1.5, −3.5]	[3, −3]	0	10	30	**04**	**05**	**28**	07	01	74	07	01	55	42	37	02
ii	[1.5, −3.5]	[2.5, −3.5]	10	0	70	**04**	**05**	**68**	04	05	10	02	07	46	39	40	15
iii	[−1, 4]	[2.5, −3.5]	60	40	20	19	06	68	15	10	23	37	63	10	**58**	**41**	**13**
iv	[0, 4]	[3, −3]	60	70	80	**66**	**61**	**80**	24	29	10	57	70	70	33	20	20
v	[−2.5, 3.5]	[1.5, −3.5]	70	0	10	**68**	**05**	**04**	10	05	04	46	07	02	15	40	40
vi	[−4, 1]	[0, −4]	0	70	60	**86**	**70**	**57**	27	55	71	49	32	21			
vii	[4, −1]	[3, −3]	70	80	60	85	70	70	**71**	**84**	**63**	53	28	11	65	01	32
viii	[3.5, −2.5]	[3.5, −1.5]	70	60	40	22	85	86	80	85	86	44	83	88	**75**	**50**	**51**

a Examples of eight types of trimers (i–viii) from Fig. 3e. The displacement vectors *v⃗*_*i*,*j*_ between molecules *i* and *j* and the molecular orientations β_*i*_ relative to the substrate lattice were determined from experimental data. The estimated uncertainty of the orientation angles is ±5°. Owing to the symmmetry of the molecules, ±90° may be added to any β_*i*_. Calculated orientations are listed for the global minimum of the total energy (state 0) and the closest local minima (states 1 to 3). The surface interaction Φ_*s*_ was scaled using γ = 0.3. The calculated orientations matching the experimental observations are marked in bold face.

To construct a model of the intermolecular interactions, we performed DFT calculations of a *v⃗* = [0, −4] dimer, *d* = 1.41 nm, on Pb(100) to get a handle on the molecule–substrate interaction. Here (Figure 3f, left panel), the top molecule was fixed at β_1_ = 18°, while the bottom molecule was allowed to relax. In the optimized geometry (Figure 3f, right panel), the lower molecule has rotated to β_2_ = 35°. This geometric change increases the distances between the hydrogen atoms. Furthermore, a C–H bond of one molecule points toward a peripheral nitrogen atom of the other molecule (dashed ellipse in Figure 3f) which resembles the orientation in self-assembled monolayers of PbPc and H_2_Pc and increases the polarization of the C–H bond.^30,31,33^

Figure 3g shows that variation of β rapidly increases the dimer energy (black curve). A twist by ≈5° raises the energy above the rotation barrier height of 56 meV of the single molecule. Consequently, rotation of the lower molecule is transmitted to the upper molecule, as well. The energy variation is largely due to the direct intermolecular interaction (blue) and is little affected by the molecule–substrate interaction (magenta).

**Figure 4:**
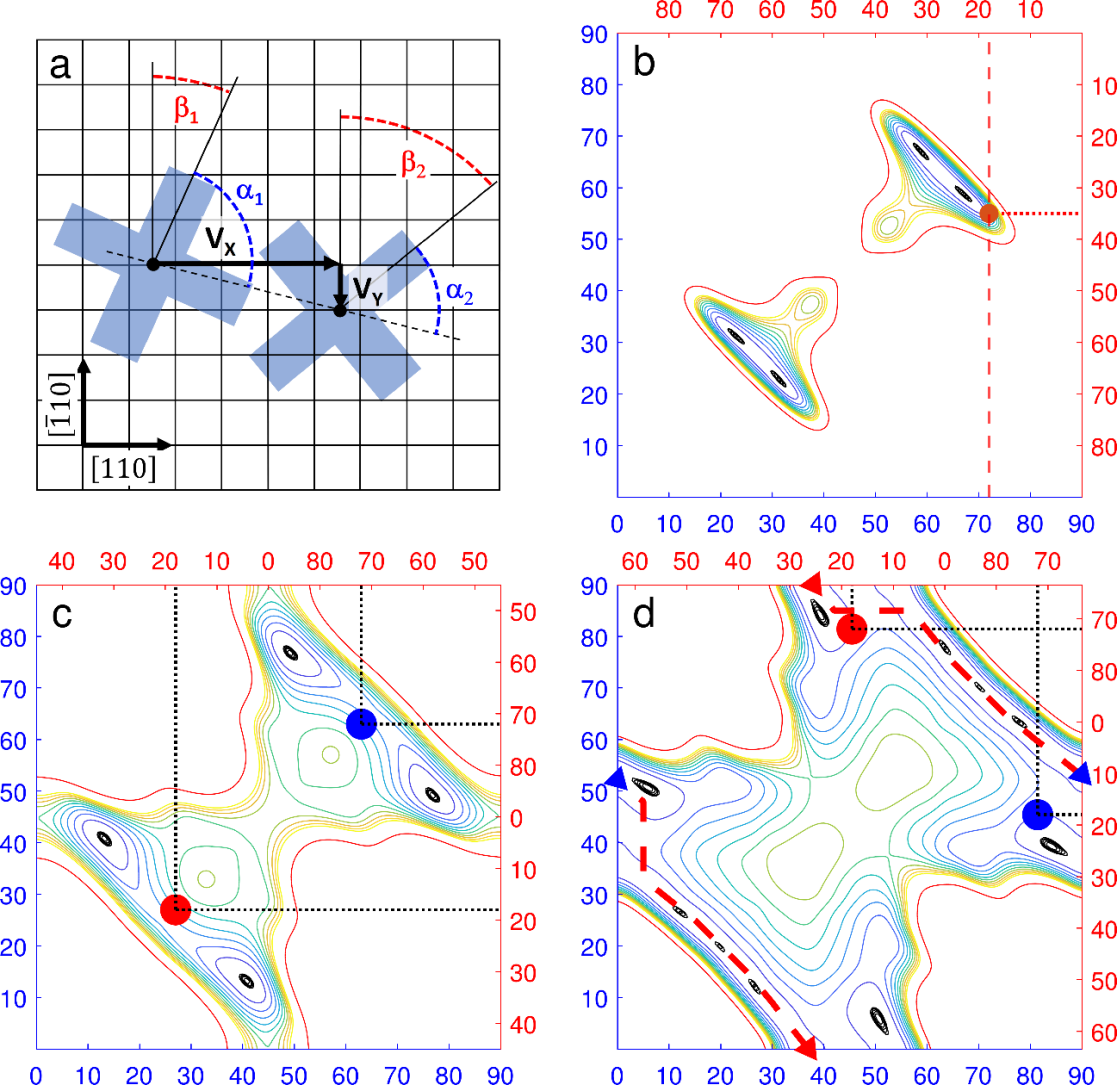
**Pair interaction between two rotors. a,** Definition of angles and displacement vectors. *v⃗* (in this case [4, −1]) denotes the vector connecting two molecules. β_*i*_ is the angle (modulo 90°) between an isoindole lobe of molecule *i* and a substrate ⟨110⟩ direction. α_*i*_ denotes the angles between the dimer axis and an isoindole lobe of molecule *i*. **b–d**, Pair potentials Φ_*p*_(α_1_, α_2_) for dimers with *d* = 1.40, 1.44, and 1.57 nm (*v⃗* = [4, 0], [3, 3] and [4, 2]) in **b**, **c**, and **d**, respectively, derived from gas-phase DFT calculations. Blue (red) axes correspond to α_1,2_ (β_1,2_). Contour lines are separated by 5 meV and range from 0 (black) to 50 meV (yellow). A red contour indicates a pair potential of 100 meV. The dashed red line in **b** illustrates a fixed β_1_ = 18° for which the structure optimization of Figure 3f was performed, and the red dot marks the relaxed orientation β_2_ = 35°. In panels **b** and **c**, the global minima are situated at α_1_ – α_2_ = ±8° and ±18°. The red and blue dots in panels **c** and **d** mark the experimentally observed vectors [3, 3] (Figure 2e,f) and [4, 2] (Figure 2a,b) dimers. In the geometry shown in panel **d**, a continuous collective rotation of both molecules along the red dashed line (barrier ≈ 7 meV) is feasible.

To explore the intermolecular interactions over a wider range distances and relative orientations, we used gas-phase DFT calculations. Interaction with the substrate is initially neglected. The resulting potential Φ_*p*_(α_1_, α_2_, *d*) is expressed in terms of the internal coordinates introduced in Figure 4a, namely the distance *d* and the angles α_*i*_ between the dimer axis and an isoindole lobe of each molecule *i*. Contour plots of Φ_*p*_(α_1_, α_2_) are shown in Figure 4b–d for three distances *d* = 1.40, 1.48, and 1.57 nm.

At the shortest distance (Figure 4b), the potential is minimal at α_1_ = 21° and α_2_ = 33° or vice versa, which corresponds to a 12° twist between the molecules. There is hardly any barrier between these minima (height ≈ 5 meV). Equivalent minima are found at 90°−α_*i*_ due to the 4-fold symmetry of the molecule and the mirror symmetry along the dimer axis. For *d* = 1.48 nm, the twist between the molecules increases to 27°, the optimal α_*i*_ are 14° and 41°, and the barrier remains low (≈20 meV). Although the low barrier allows for transitions between both configurations, a correlated rotation beyond 90° is prevented by a large barrier indicated by the 100 (yellow) and 500 meV contours (red). At *d* = 1.57 nm, this barrier collapses allowing for a continuous rotation of both molecules in opposite directions. This cogwheel-like motion is indicated by a red dashed path in Figure 4d and exhibits a minor barrier of ≈10 meV.

### Application of the Pair Potential to Trimers

**Figure 5:**
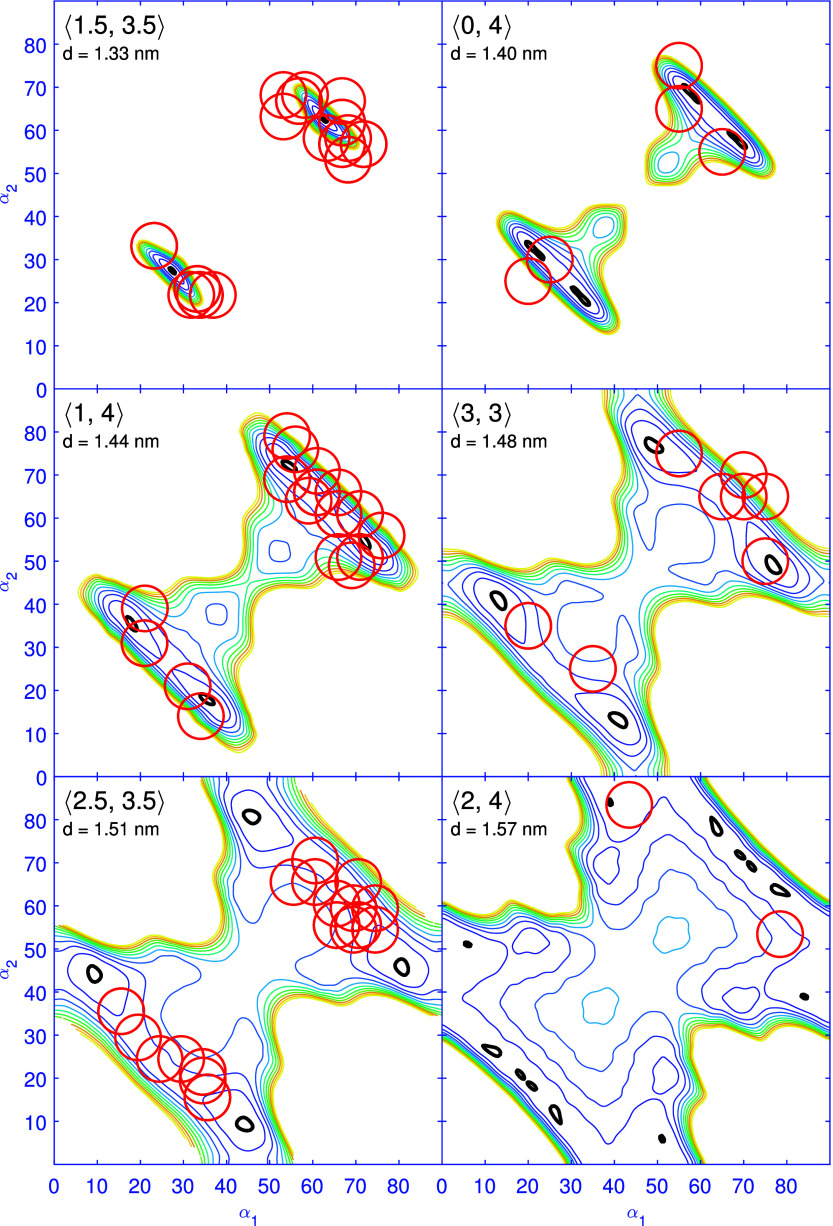
**Model potentials and measured orientations in trimers.** Pair potentials Φ_*p*_(α_1_, α_2_) for intermolecular distances *d* as indicated in each panel. Symmetry-equivalent orientations of the dimers have been merged. That is to say, the connecting vectors [*x*, *y*], [*x*, −*y*], [*y*, *x*], [*y*, −*x*] are not discriminated and are collectively denoted ⟨*x*, *y*⟩. The energy contours are separated by 10 meV and range from 1 (thick black line) to 100 meV (yellow line). The red circles indicate the pairs of angles observed in a set of 51 trimers, and the diameter of each circle indicates the estimated uncertainty of the measured angles.

We analyzed the data from 52 trimers with different orientations and separations of the AlPc molecules. The observed molecular orientations α_1_ and α_2_ for each pair of adjacent molecules are displayed in Figure 5, which also shows equipotential lines of the calculated pair interaction Φ_*p*_. Coordinate pairs α_1_, α_2_ are equivalent to 90°−α_1_, 90°−α_2_ by mirror symmetry at the dimer axis as can bee seen in Figure 4a.

Despite the simplicity of the model and the neglect of the substrate interaction, the agreement of the measured pairs of angles with the predictions is rather good. All observed angles are located in low energy regions of the potential landscape, demonstrating the decisive role of the pair interaction.

At the shortest separations observed (*d* = 1.33 nm, Figure 5), the potential enforces a single well-defined angle α_1_ = α_2_ = 27° (or the mirror symmetric equivalent 63°). As *d* successively increases (panels b–f), each minimum splits into a pair α_1_, α_2_ and α_2_, α_1_. These minima correspond to the bistable orientations observed in e.g., Figure 2a and b. The twist between the molecules, |α_1_ – α_2_|, also increases with *d* because the steric hindrance via H ions decreases and the polarization of a C–H bond is enhanced. Starting from panel *d* = 1.48 nm, the 100 meV contour is no longer closed, implying that the barrier for a continuous rotation of both molecules in opposite directions (α_1_, α_2_ → α_1_ + δ, α_2_ – δ) begins to collapse.

We now combine the gas phase pair potential Φ_*p*_(α_*i*_, α_*j*_, *d*) and the rotation barrier on the substrate Φ_*s*_(β_*i*_), to a total energy *E*
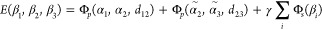
The vectors *v⃗*_12_ and *v⃗*_23_ connecting the centers of the molecules in trimers are usually not collinear, and consequently, two internal angles α_*i*_ and α̃_*i*_ referring to these vectors are used. To cope with the DFT-related uncertainty of Φ_*s*_ mentioned above, a scaling factor γ is introduced.

A lower limit of γ may be determined from the [3, 3] dimer of Figure 2e, where β = 18° and α = 27° for both molecules. As shown in Supporting Figure S4, minima of the potential *V* are located at α_1_ = 14° and α_2_ = 41° (and angles equivalent by symmetry) when the molecule–substrate interaction is neglected (γ = 0). They move toward the experimental value of α_1_ = α_2_ = 27° when γ exceeds 0.2. In other words, the substrate has some impact on the dimer geometry, but as will be shown below, the effect is smaller than calculated, i.e., γ < 1.

For each trimer of Figure 3e, the total energy *E* was calculated for varying the γ. Table 1 lists the angles of local minima (States 0–3) for γ = 0.3. In all cases, one of the calculated states (marked in bold face) agrees with the experimental angles within the experimental uncertainty of ±5°. Whenever two molecules inside a trimer are very close (*d* = 1.33 nm, vectors ⟨1.5, 3.5⟩, trimers i, ii, v, and viii in Table 1), the model predicts two nearly identical orientations because Φ_*p*_ has a single minimum at α_1_ = α_2_ at this distance. Experimentally a mean twist |α_1_ – α_2_| ≈ 11° is observed. This may indicate an inaccuracy of the pair potential from gas-phase DFT.

As several local minima are predicted, different orientations of the molecules within the trimers should be stable. Indeed, we observed trimers with identical *v*_12_ and *v*_23_ but different orientations of the molecules. For example, trimers of type viii were observed with orientations (β_1_, β_2_, β_3_) = (70°, 60°, 40°) and (20°, 90°, 80°). Similarly, types ii (Supporting Figure S5), iv, and v have been observed in state 0 and also with (45°, 30°, 20°), (20°, 30°, 20°), and (20°, 30°, 40°), respectively, which match the states 3, 1, and 3 of the model within a 10° margin.

When γ is increased, minor changes in the predicted orientations occur. The energies of the local minima with orientations β_*i*_ that deviate more strongly from the minima of Φ_*s*_ at β = ±18° are increased proportional to γ. For example, states 3 of trimers iii and viii, which are closest to the experimentally observed orientations, are global minima at γ = 0 and shift up in energy by Δ*E* ≈ γ · 120 meV. The agreement between model and experiment thus deteriorates when γ ≳ 0.5.

One possible source of the deviation of γ from 1 is the inaccuracy of the rotation barrier height from DFT. In addition, the presence of the STM tip leads to attractive dispersion interaction,^16,39,40^ which lifts the molecules and thus reduces the barrier height. Indeed, an increase of the molecule–substrate separation by 10 pm reduces the calculated barrier height by ≈10 meV.

The AlPc on the Pb(100) system permits the formation of unconventional arrangements including loops with increasing voids. Supporting Figure S6 illustrates examples comprising six, seven, eight, and nine AlPc molecules. In addition to the angles observed in the trimers, other values of β are stabilized in these loops. In these arrays, each molecule is fixed in position by two neighboring molecules, preventing collective rotation.

The orbital energies and spin states of molecules in a two-dimensional array are dependent upon the number of neighboring molecules and their orientations, as was previously observed in 3 × 3 arrays of phthalocyanines.^29,30,33,41^ The molecular loops studied here display the same effect as illustrated in Supporting Figure S7.

In conclusion, isolated AlPc molecules are observed to adsorb on the bridge sites of the Pb(100) surface. Their lateral orientation is found to be unstable, as evidenced by an unusual intramolecular contrast of 16 lobes. DFT calculations indicate the presence of a low barrier height for rotation when a fairly thick Pb slab is employed. Molecular dimers and trimers were assembled with the STM. Excitation of one of the components has been demonstrated to be an effective means of inducing collective rotation. Modeling with a DFT-derived pair potential reproduces a large number of observed molecular orientations. The azimuthal orientation of the molecules is controlled by mechanical interlocking and hydrogen bonding, while the influence of the substrate is less pronounced in this system. The use of classical multidimensional potentials may be a valuable approach in investigations of large molecular structures that are currently beyond the scope of ab initio descriptions.

## Methods

Experiments were performed with a scanning tunneling microscope operated at a temperature of 4.2 K under an ultrahigh vacuum. Pb(100) surfaces were prepared by cycles of Ar ion bombardment (1.5 keV) and annealing to ≈530 K. Chloroaluminum phthalocyanine molecules were sublimated from a heated crucible onto the substrate at ≈300 K. The sample was then annealed at 470 K for 5 min. STM tips were electrochemically etched from W wire and annealed *in vacuo*.

## Data Availability

The data supporting the findings of this study are available from the corresponding author upon request.

## Supporting Information Available

Theoretical details; calculations of the adsorption geometry; determination of the scaling factorγ; alternative trimer configurations; construction of molecular loops; spectroscopic data from a molecular loop (PDF)
