# Supplementary material for: Mechanically Interlocked Molecular Rotors on Pb(100)
Source: Nano Lett. 2025 Jan 13;25(4):1504–11. doi: 10.1021/acs.nanolett.4c05409 (PMC11783589; doi:10.1021/acs.nanolett.4c05409)
Supplement: Supplementary file 1 — nl4c05409_si_001.pdf [file nl4c05409_si_001.pdf]

Supporting Information for: Mechanically Interlocked Molecular Rotors on Pb(100)

Chao Li,[†] Yan Lu,[‡] Ruoning Li,[¶] Li Wang,[‡]

Alexander Weismann,[*,†] and Richard Berndt[†]

†*Institut für Experimentelle und Angewandte Physik, Christian-Albrechts-Universität, 24098 Kiel, Germany*

‡*Department of Physics, Nanchang University, Nanchang 330031, People's Republic of China*

¶*Key Laboratory of Molecular Nanostructure and Nanotechnology and Beijing National Laboratory for Molecular Sciences, Institute of Chemistry, Chinese Academy of Sciences (CAS), Beijing 100190, People's Republic of China*

E-mail: weismann@physik.uni-kiel.de

Experimental Methods

Experiments were performed with a scanning tunneling microscope operated at a temperature of 4.2 K in an ultrahigh vacuum. Pb(100) surfaces were prepared by cycles of Ar ion bombardment (1.5 keV) and annealing to $\approx$ 530 K. Chloroaluminum phthalocyanine molecules were sublimated from a heated crucible onto the substrate at $\approx$ 300 K. The sample was then annealed at 470 K for 5 minutes. STM tips were electrochemically etched from W wire and annealed *in vacuo*.

Theoretical Details

Calculations were performed using density functional theory (DFT) as implemented in the Vienna ab initio simulation package (VASP),[1,2] with the Perdew-Burke-Ernzerhof (PBE) functional.[3] A plane-wave basis set with a kinetic energy cutoff of 400 eV was adopted for geometry optimization and self-consistent calculations. Eleven layers of Pb atoms, separated by an 18 Å vacuum region, were used to model the Pb(100) surface. For geometry optimizations, all atoms, except the bottom five Pb layers were relaxed until the residual force per atom was less than 0.02 eV/Å. Van der Waals (vdW) interactions were treated using the the DFT-D3 method.[4] The Brillouin zone was sampled at the $\bar{\Gamma}$ point.

Calculations of the Adsorption Geometry

The experimental data showed that single AlPc molecules adsorb at bridge sites of Pb(100) with azimuthal angles of 18° and 72°. In our DFT calculations eleven layers of Pb were required to reproduce the preference for bridge site adsorption (Fig. S2) while top sites were preferred in calculations with only six layers (Fig. S1). We tentatively attribute the large number of substrate layers required to strong quantum size effects in the Pb slabs.[5–7]

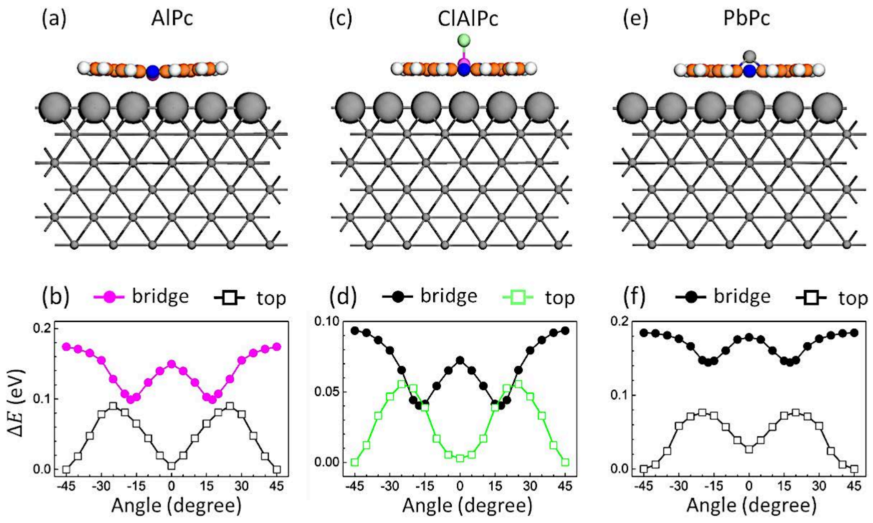

Supporting Figure 1: DFT results for a 6-layer slab of Pb(100). The atoms of the bottom three layers were fixed at their bulk positions. (a, c, e) Side views of AlPc, ClAlPc, and PbPc. (b, d, f) Corresponding energies at bridge and top sites.

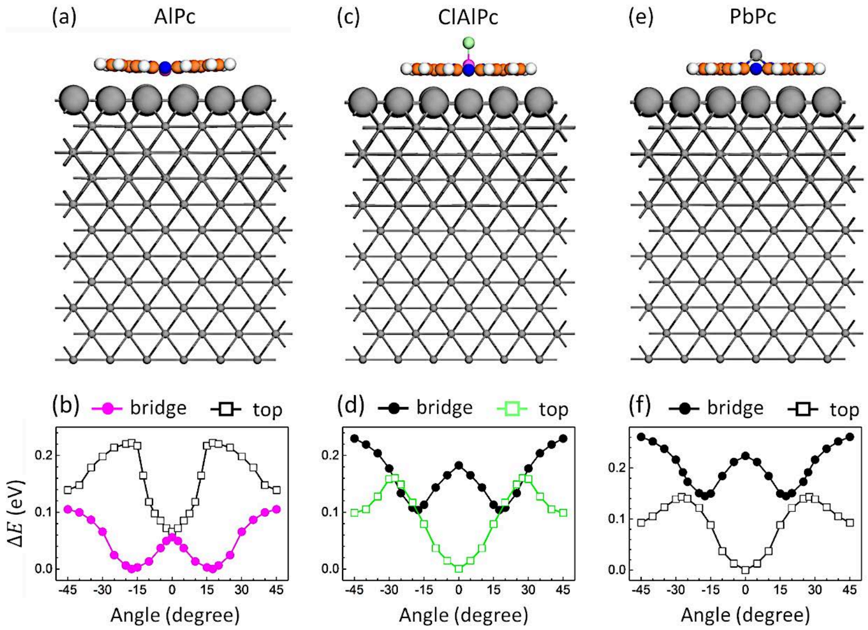

Supporting Figure 2: DFT results for an 11-layer slab of Pb(100). The atoms of the bottom five layers were fixed at their bulk positions during geometry optimization. (a, c, e) Side views of AlPc, ClAlPc, and PbPc. (b, d, f) Corresponding energies at bridge and top sites versus the angle between the isoindole lobes and a substrate ⟨110⟩ direction.

Calculation of the Translation Barrier

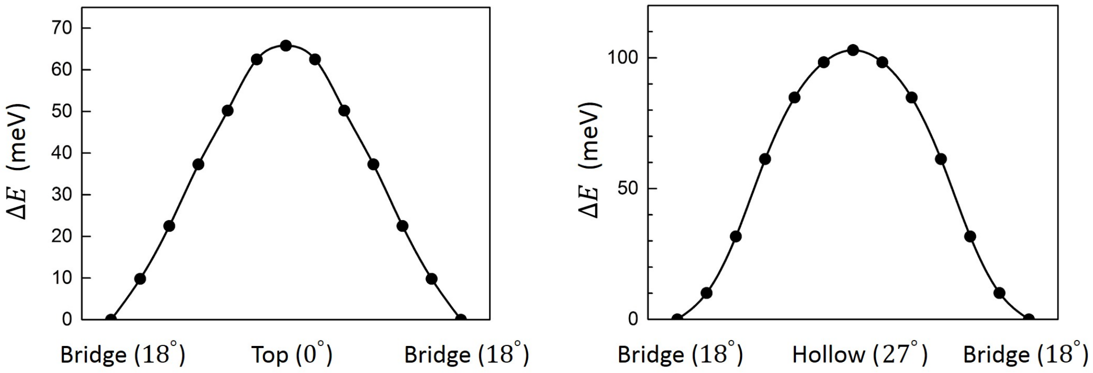

Supporting Figure 3: Energy barriers for molecular translation between adsorption sites calculated using DFT. The molecule was allowed to rotate during the translation. The left panel shows the translation between bridge and top sites, while the right panel illustrates the translation via hollow sites. The energy barrier for translation from a bridge site to a top site (hollow site) is approximately 10 (50) meV higher than the rotational barrier at bridge sites.

Determination of the Scaling Factor γ

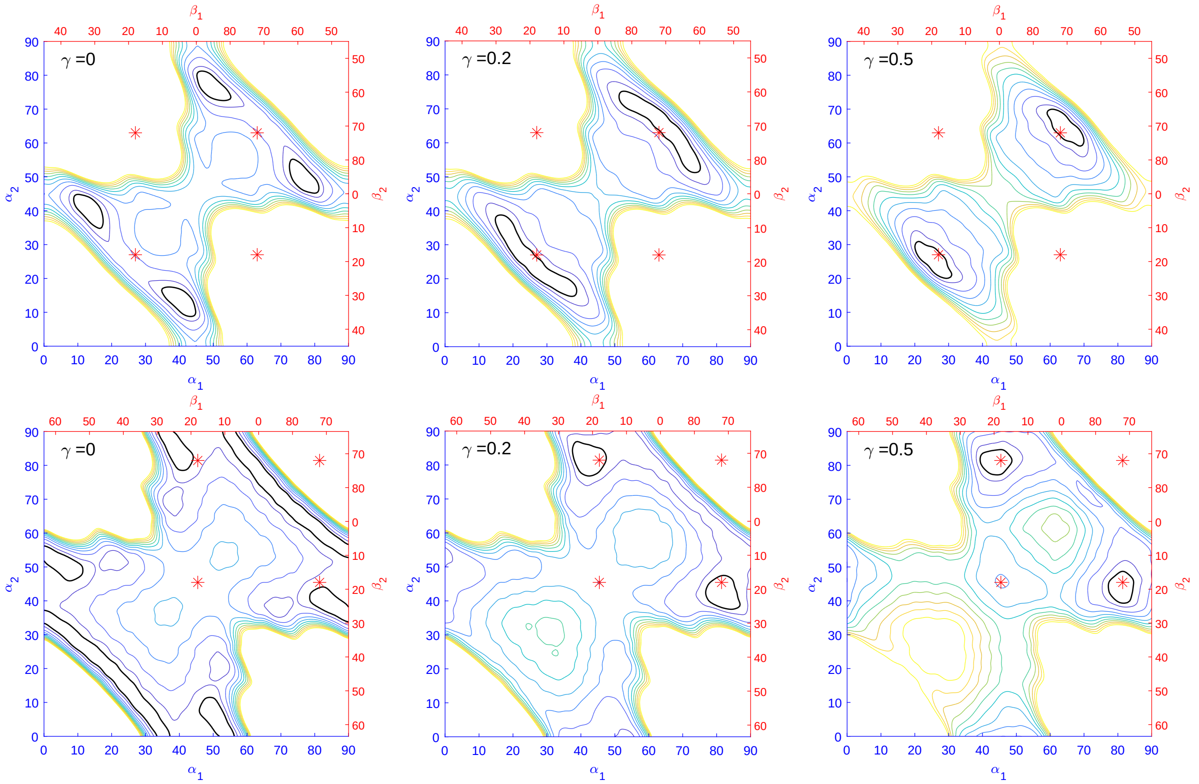

Supporting Figure 4: Potential energy landscapes of [3, 3] (top) and [4, 2] (bottom) dimers for different scaling factors γ of the molecule-substrate interaction. Red asterisks indicate the minima of Φ_s at $\beta = \pm 18°$. Contours range from 5 (black) to 100 meV (yellow). For the [3, 3] dimer, $\gamma > 0.2$ is required to stabilize the geometry at $\beta_1 = \beta_2$, while for the [4, 2] dimer the experimentally observed angles $\beta_1 = -\beta_2$ are close to a local minimum of the shere pair potential, *i. e.* at $\gamma = 0$.

Alternative Trimer Configurations

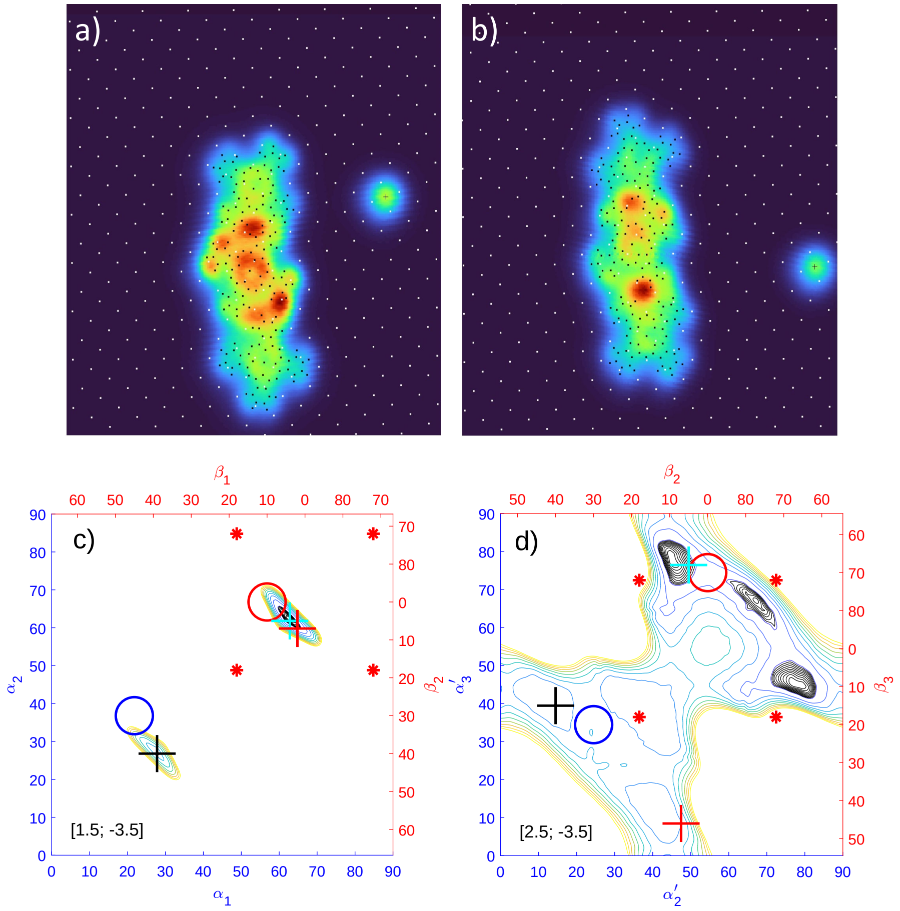

Supporting Figure 5: Example of two trimer configurations found for the same displacement vectors $\vec{v}_{12} = [1.5, -3.5]$ and $\vec{v}_{23} = [2.5, -3.5]$. a) STM image (100 mV, 100 pA) of a trimer with $\beta = (10°, 0°, 70°)$ similar to trimer ii of the manuscript. The atomic lattice of the substrate is indicated by white points. A single atom on a hollow position (+) was used as a landmark. The structure of the molecules is indicated by black points. b) Same for a different trimer with $\beta = (45°, 30°, 20°)$. c) and d): Potential energy with $\gamma = 0.3$. Contours range from 10 (blue) to 100 meV (yellow) in steps of 10 meV. Black contours range from 1 to 9 meV in steps of 1 meV. Red and blue circles indicate the experimental angles of the trimers shown in a) and b), respectively. Cyan, red and black crosses show the calculated states 0 (4°, 5°, 68°), 2 (2°, 7°, 46°) and 3 (39°, 40°, 15°). Red asterisks indicate the minima of Φ_S.

Construction and Spectroscopic Data from Molecular Loops

The data in Fig. S6 and Fig. S7 show that loops of interlocked molecules are sufficiently stable to enable further manipulations and spectroscopic measurements. After constructing a 9-molecule loop with a tenth molecule inside, another molecule was attached at various positions on the outer circumference of the loop (Fig. S7(a – d)). Spectra recorded from the lobes of some molecules are shown in panel (e). The presence of a localized spin on the probed molecules is signaled in-gap states with different peak heights at positive and negative bias polarity and a local minimum outside the YSR resonances. The purple curve (the position of the measurement is indicated in panel a) shows that the proximity of three nearest neighbors is not sufficient to induce a paramagnetic state. In contrast, some molecules with four nearest neighbors, shown in panels b and d, exhibit YSR states with $\Delta_T + E_{YSR} = 2.50$ and $2.45\,\mathrm{mV}$, respectively, as illustrated in panel (e) by the blue and yellow curves. In absence of YSR states (purple and red curves), conductance peaks are observed at $\pm(\Delta_T + \Delta_S) = \pm 2.55\,\mathrm{mV}$.

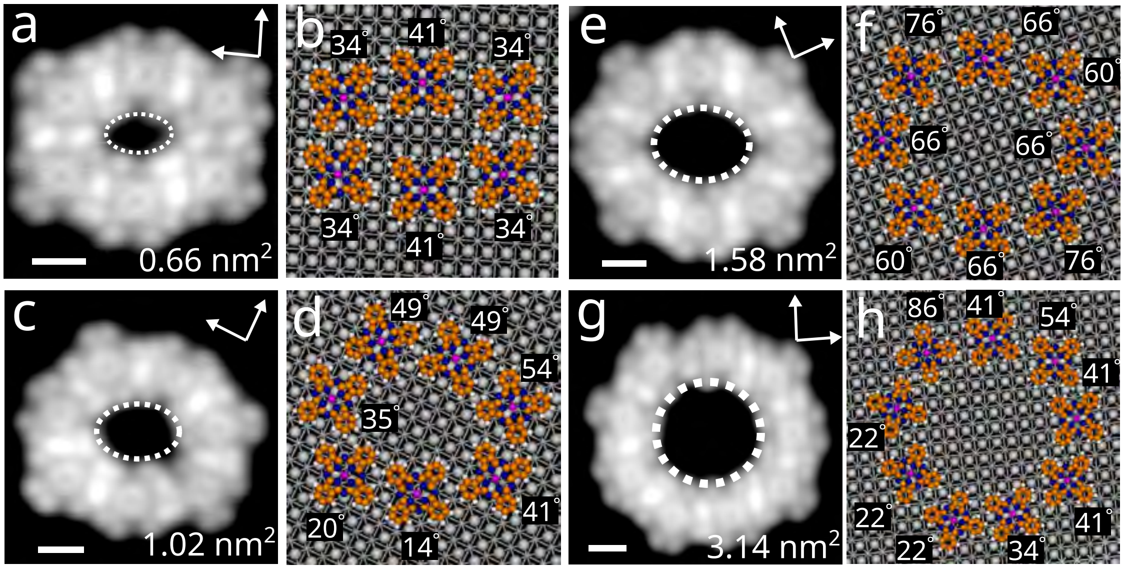

Supporting Figure 6: **Artificial molecular loops. a, c, e, g** Constant-current images of rings with six, seven, eight, and nine molecules made by manipulation with the STM tip. The areas of the voids are indicated. The orientations of the molecules are indicated by crosses. Large apparent heights at interstitial positions are not attributable to some form of bond contrast; rather, they are a consequence of the incoherent superposition of molecular states that are situated well above the molecular plane. **b, d, f, h** Corresponding models. Angles β shown around the molecules. Imaging parameters: $I = 100\,\text{pA}$; $V = 100$, 120, 100, and 180 mV in a, c, e, and g, respectively.

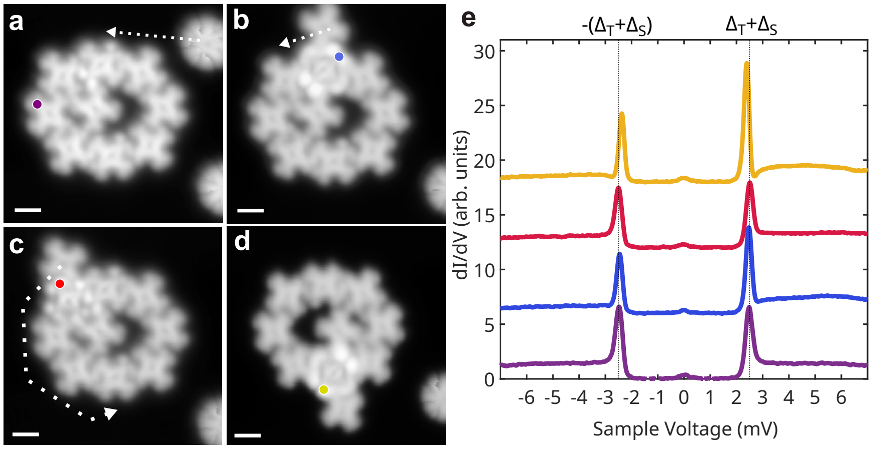

Supporting Figure 7: (a – d) Topographs (10 mV, 100 pA) of a 9-molecule loop with a tenth molecule inside made by manipulation with the STM tip. An additional molecule that is isolated in (a) is successively moved along the outer circumference of the loop in (b – d) as indicated by the arrows. From (c) to (d), the molecule inside the loop was also moved. (e) Low-bias dI/dV spectra ($V_{mod} = 30\,\mu\text{V}_{\text{PP}}$ at 831 Hz) recorded at the positions indicated on the topographs in matching colors.

Supporting references

1. Kresse, G.; Furthmüller, J. Efficient Iterative Schemes for Ab Initio Total-Energy Calculations using a Plane-Wave Basis Set. *Phys. Rev. B* **1996**, *54*, 11169.

2. Kresse, G.; Joubert, D. From Ultrasoft Pseudopotentials to the Projector Augmented-Wave Method. *Phys. Rev. B* **1999**, *59*, 1758.

3. Perdew, J. P.; Burke, K.; Ernzerhof, M. Generalized Gradient Approximation Made Simple. *Phys. Rev. Lett.* **1996**, *77*, 3865.

4. Grimme, S.; Antony, J.; Ehrlich, S.; Krieg, H. A Consistent and Accurate Ab Initio Parametrization of Density Functional Dispersion Correction (DFT-D) for the 94 Elements H-Pu. *J. Chem. Phys.* **2010**, *132*.

5. Yu, D.; Scheffler, M.; Persson, M. Quantum Size Effect in Pb (100) Films: Role of Symmetry and Implications for Film Growth. *Phys. Rev. B* **2006**, *74*, 113401.

6. Han, Y.; Liu, D.-J. Quantum Size Effects in Metal Nanofilms: Comparison of an Electron-Gas Model and Density Functional Theory Calculations. *Phys. Rev. B* **2009**, *80*, 155404.

7. Li, W.; Huang, L.; Pala, R. G. S.; Lu, G.-H.; Liu, F.; Evans, J. W.; Han, Y. Thickness-Dependent Energetics for Pb Adatoms on Low-Index Pb Nanofilm Surfaces: First-Principles Calculations. *Phys. Rev. B* **2017**, *96*, 205409.
